# Infrared thermography reveals weathering hotspots at the Požáry field laboratory

**DOI:** 10.1038/s41598-024-65527-x

**Published:** 2024-06-25

**Authors:** Marco Loche, Ondřej Racek, Matěj Petružálek, Gianvito Scaringi, Jan Blahůt

**Affiliations:** 1 Institute of Rock Structure & Mechanics, Czech Academy of Sciences, V Holešovičkách 41, 182 09 Prague, Czechia; 2 Institute of Hydrogeology, Engineering Geology and Applied Geophysics, Faculty of Science, Charles University, Albertov 6, 128 43 Prague, Czechia; 3 Department of Physical Geography and Geoecology, Faculty of Science, Charles University, 128 43 Prague, Czechia; 4 Institute of Geology of the Czech Academy of Sciences, Prague, Czechia

**Keywords:** Cooling rate index, Porosity, Infrared thermography, Rock mass, Slope stability, Informative time window, Natural hazards, Geology, Geophysics

## Abstract

Evaluating physical properties and mechanical parameters of rock slopes and their spatial variability is challenging, particularly at locations inaccessible for fieldwork. This obstacle can be bypassed by acquiring spatially-distributed field data indirectly. InfraRed Thermography (*IRT*) has emerged as a promising technology to statistically infer rock properties and inform slope stability models. Here, we explore the use of Cooling Rate Indices (CRIs) to quantify the thermal response of a granodiorite rock wall within the recently established Požáry Test Site in Czechia. We observe distinct cooling patterns across different segments of the wall, compatible with the different degrees of weathering evaluated in the laboratory and suggested by *IRT* observations of cored samples. Our findings support previous examinations of the efficacy of this method and unveil correlations between cooling phases in the field and in the laboratory. We discuss the scale-dependency of the Informative Time Window (*ITW*) of the CRIs, noting that it may serve as a reference for conducting systematic *IRT* field surveys. We contend that our approach not only represents a viable and scientifically robust strategy for characterising rock slopes but also holds the potential for identifying unstable areas.

## Introduction

The physical characterisation of rock samples remains foundational for identifying potential instability in rock masses prone to landslides^1,2^. The need for investigating inaccessible areas reveals the necessity for indirect approaches. In addition to mainstream practices for rock mass analysis, such as photogrammetry and LiDAR^3^, InfraRed Thermography (*IRT*) has emerged for its potential to shed light onto physical properties of rock masses. Thermal remote sensing has gained popularity steadily, transforming the systematic field approach^3–6^ with its ability to reveal micro-morphological features such as bridges^7^ and discontinuities^8^. *IRT* has proven crucial where standard field surveys are impracticable^9–12^.

Notably, *IRT* surveys offer evaluations of the Land Surface Temperature (LST)^13,14^. Indeed, LST has been identified as a proxy for properties such as porosity and parameters such as the compressive strength^12,15–17^. Therefore, landslide mapping through *IRT* campaigns has been attempted by interpreting thermal anomalies^4,18,19^, and assessments of cooling stages were found indicative of the presence of loosened material and open fractures. Advancements in sensor technology have enabled the installation of InfraRed Thermal cameras on Unmanned Aerial Vehicles, transitioning from static platform-based surveys to more dynamic assessments^11,20^. In this context, to identify areas prone to landslides, an *IRT* routine has proven suitable for both rock and soil materials^18,21^. Yet, challenges remain in this data-driven approach, particularly in determining the appropriate Cooling Rate Index (CRI)—a measure of the rate at which rock or minerals have cooled—and Informative Time Window (*ITW*)—the period of time during which a portion of a heating or cooling process takes place—which relates to the material’s properties and thermal history, and is intrinsically linked to factors of scale and heterogeneities, among others^2,7^.

Numerous dramatic events and regional statistics have drawn attention to the need to monitor vulnerable areas (e.g., highways and urban locales) susceptible to rock slope failures that could endanger lives, infrastructures, and ecosystems^22–28^. Because of the rapid evolution of such events, the demand for adequate monitoring systems has risen. In particular, efforts have been directed towards calibrating remote sensing techniques with laboratory-scale rock samples^8,15,16,29,30^. Laboratory test data have been utilised to formulate empirical correlations for application in slope and catchment-scale modelling^31–36^. The importance of identifying appropriate upscaling/downscaling strategies to produce reliable correlations even in a purely data-driven approach cannot be overstated and remains an open issue^17,37,38^.

With respect to *IRT*, specific tests have been tailored to meet heightened data collection needs^12,39–41^, thereby challenging their direct use in modelling. These studies, as aforementioned, have highlighted notable differences in LST within fractured or heterogeneous areas. To deterministically evaluate rock properties via *IRT*, a fundamental analysis of heat transfer from rocks to the atmosphere would be necessary, which could target the cooling phase post-attainment of the daily maximum temperature. This consideration poses primary challenges owing to varying outcrop and sample dimensions and internal heterogeneities, potentially leading to incongruent results. Addressing this, research on scale factor assessment has arisen to translate laboratory outcomes into larger areas^42,43^. From an empirical perspective, the CRI, calculated both in the laboratory and the field on the same lithological type, may play a key role in evaluating the superficial evolution of the rock and its relationship with weathering processes. The cooling rate of a rock mass depends on its mineralogical composition, porosity, texture, and weathering properties. Notably, minor inaccuracies can arise from changes in emissivity and camera angle, yet field examples and laboratory tests can identify spatial temperature differences typically well exceeding the magnitude of errors^44,45^.

In this study, we present experiments conducted on a granodioritic rock wall and on several samples cored from it, employing *IRT* in tandem with a physico-mechanical characterisation. Through regression analysis, we establish empirical relationships between the CRI evaluated for different time intervals and properties of rock samples with various degrees of weathering (including porosity, Young’s and shear moduli, and Mode I fracture toughness), whereby the main stress was on confirming the possible linear relationship with porosity. However, the objective of this study extends beyond identifying the best-fit relationship in laboratory and field settings as we foresee a plausible approach to leverage the insights collected from laboratory tests to estimate the cooling dynamics of real slopes. This could be achieved in future work by performing an extensive selection of CRIs for the field and subsequently identifying a scaling factor to standardise the outcomes, aided and complemented by a physically-based understanding and modelling of the cooling process.

The use of the *ITW* is suggested to identify the optimal time interval for CRI analyses. In fact, the *ITW* relies on the identification of a representative secant slope in the cooling curve, which carries time-averaged information on the physical process of heating or cooling of the rock, thereby attributing more physical meaning to the CRI compared to evaluation based on the time-derivative of temperature (cooling rate). Also, once the *ITW* is established, it may allow quicker determinations, eliminating the need to track the entire cooling curve or fully characterise it if a purely data-driven approach is pursued.

## Methods

### Case study

**Figure 1:**
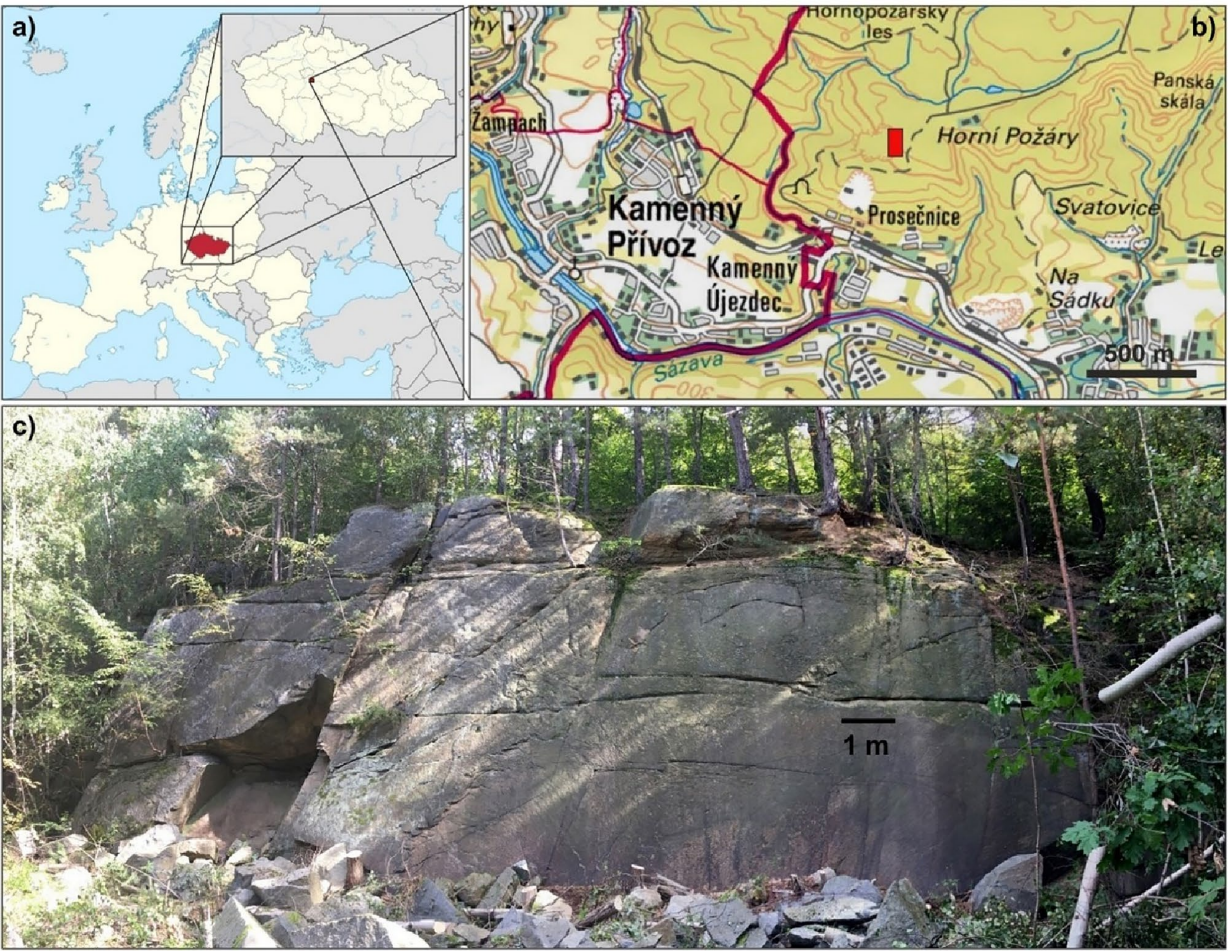
(**a**) Location of the study area in Czechia, Europe; (**b**) the Požáry Test Site is identified by a red rectangle (map sources: Wikimedia Commons and Czech Cadastral Office); (**c**) overview of the west-facing rock wall before the installation of the monitoring system. The maps were produced in ArcMap v. 10.8 (ArcGIS Desktop, ESRI, https://www.esri.com/en-us/home); post-processing was done in CorelDRAW 2019 (https://www.coreldraw.com/); the photograph of the site was taken by O. Racek.

The research was conducted at the Požáry Test Site in central Czechia (Fig. 1). The site, once a biotitic granodiorite quarry, forms part of the Central Bohemian Plutonic Complex^46^. A segment of the quarry currently serves as a principal point for scientific investigation, with the establishment of a comprehensive monitoring system. The prevalent rock within the region exhibits relative uniformity, featuring medium-grained texture (0.5–2 mm) and a mineral composition comprising quartz (33%), K-feldspar (15%), plagioclase (48%), and biotite (4%)^47,48^. The goal within this area is to track alterations in rock slope behaviour and ascertain the impact of climate change on the stability of the rock mass^49–51^. To do so, the monitoring system encompasses a meteorological station, crack meters, strain gauges, and a thermometer designed to measure temperature within the rock mass, spanning from the surface to a depth of 3 m^52^. The rock face, while notably uniform and vertical, stands at approximately 8.6 m in height and spans 21.5 m horizontally, with a western orientation. Nonetheless, on a broader scale, a fracture network is visible with horizontal and vertical discontinuities (Fig. 1). These differences could arise from a combination of factors, including weathering, tectonic activity, and historical blasting during the quarrying process. Such interactions may contribute to the development of weathered zones and pathways for erosion within the fracture network.

### Preliminary monitoring of the rock mass

**Figure 2:**
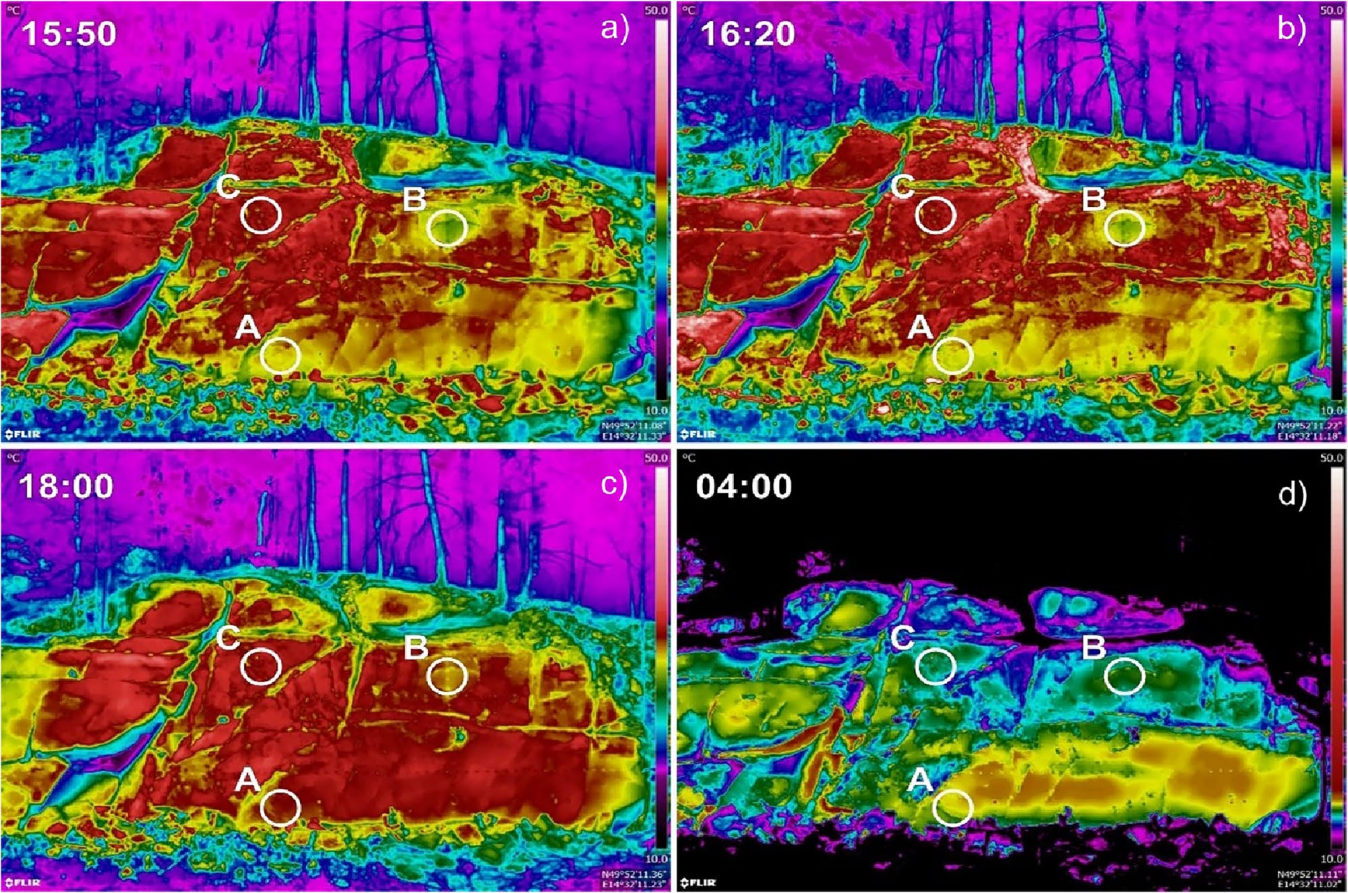
Thermograms were recorded at the slope scale where substantial thermal anomalies appeared, suggesting different behaviours within the rock mass. ROIs were selected based on the visual anomalies of the acquired images.

To study the thermo-mechanical evolution of the rock wall, an *IRT* time-lapse was performed from 12:00 UTC on 13 May 2022 to 12:00 UTC on 14 May 2022, capturing images each 10 min, following a previously established practice that has proven appropriate^12,15^. For this purpose, a FLIR E95 thermal camera—with a resolution of 464 × 348 pixels at 30 Hz, a spectral range of 7.5–14 μm, and equipped with a 42° lens—was fixed to a tripod positioned 40 m from the rock mass, resulting in an average geometrical resolution at the pixel scale of 74 mm, following a monitoring strategy outlined in the literature^45,52^. Given the absence of apparent differences in the exposure/orientation of the rock wall, the *IRT* campaign was designed to assess the thermal behaviour of multiple Regions Of Interest (*ROIs*). Remarkably, the daily time-lapse analysis unveiled three predominant regions manifesting distinct thermal behaviours (see video time-lapse in the supplementary material), as summarised in Fig. 2. In the figure, acquisition times were as follows (in UTC): (a) 15:50, warming peak or the beginning of CRI calculation; (b) 16:20, CRI30min (i.e., calculated over 30-min intervals) used in the correlation calculations; (c) 18:00, CRI inflexion point; (d) 04:00, cooling valley. The samples were collected in areas of the rock wall with not particularly different orientations and superficial aspects. The subdivision into compact rock (Sample A), compact but divergent thermal behaviour (Sample B), and visibly weathered rock (Sample C) was confirmed by laboratory tests. On this basis, the suggestion of distinct rock mechanical conditions gained validation from the correspondence of thermal behaviours within the ROIs and laboratory tests (see also Fig. S1).

To understand the reasons behind the varying behaviours observed within such a uniform micro-topography, we focused our analyses on the *ROIs* specifically. These (termed Sample A, B, C) were selected, using a circular outline, on the basis of expert judgment on the observable thermal anomalies within the acquired *IRT* time-lapse images. Within them, the temperature signal remained reasonably stable following their own temperature pattern. Following data acquisition, post-processing was conducted using FLIR ResearIR software (FLIR Systems, Inc. Wilsonville, OR, USA). Each image underwent correction based on factors including the distance to the object, emissivity, air temperature, and relative humidity, collected from the meteorological station located on the rock wall. Emissivity was fixed at 0.96 according to the material characteristics, and the variability in the parameter exhibited no notable deviations in results, as also outlined by Sass et al.^45^.

### Post-processing of field thermal images

To quantitatively assess the thermal dynamics of the *ROIs*, we used the CRI, which quantifies the change in mean temperature (∆T, °C) per unit of time (∆t, min). Higher CRI values correspond to more rapid cooling of the rock mass for a given time interval, as summarised in Eq. (1)^12,15,39^:

1 $$ {\text{CRI }} = \, \Delta {\text{T}}/\Delta {\text{t}}{.} $$

The cooling trends and, subsequently, the CRI outcomes exhibit their informative significance and lead to the assessment of the optimal *ITW*, which serves as a crucial element for effective monitoring and systematic surveying.

### Preliminary characterisation of the rock samples

Based on the thermal assessment of the sectors, samples obtained through coring within the *ROIs* A, B, and C were studied. The samples produced by the coring in these sectors are revealed to correspond to three stages of weathering exhibited by rock masses. Sample A, characterised by its intact nature devoid of microcracks and visible weathering signs, is classified as fresh rock. Sample B, showing microcracks yet lacking macroscopic weathering indications, is identified as compact rock. Sample C, marked by partially disintegrated rock with macrocracks and microcracks, is labelled as heavily weathered or altered. These classifications adhere to weathering grades I (fresh rock), II (slightly weathered rock), and II-IV (moderately to highly weathered rock), according to Hatheway^53^.

Further, we conducted laboratory experiments to quantify physical parameters and obtain an accurate characterisation. A non-destructive categorization encompassing grain density, dry density, porosity, P-wave velocity, S-wave velocity, and dynamic elastic moduli estimated from P- and S-wave velocities was executed. Subsequently, Mode I fracture toughness (KIC) was estimated on the very same samples. These tests adhered to ISRM-suggested methodologies^53^ for dynamic elastic properties^54^ and Mode I fracture toughness^55^. Table 1 and Fig. S2 present the mean values alongside their corresponding standard deviations, derived from laboratory tests. Notably, the non-destructive tests were performed on specimens initially intended for the KIC test, as explained in Hatheway^53^, thereby consistently characterising the behaviour of selected samples and *ROIs*. In fact, the KIC test allowed to produce diverse samples which are representative of the superficial face of the rock.

**Table 1:** Physical and mechanical properties of the samples: grain density (ρS); dry density (ρD); total porosity (n); P-wave velocity (vP); shear wave velocity (vS); Young moduli (Ed); Poisson ratio (vd); Shear moduli (ud); Bulk moduli (Kd); mode I fracture toughness (K_IC_).

Sample	A		B		C	
	µ	σ (%)	µ	σ (%)	µ	σ (%)
ρS (g/cm^3^)	2.667	0.1	2.662	0.1	2.649	0.2
ρD (g/cm^3^)	2.639		2.608		2.550	
n (%)	0.90		1.69		3.75	
vP (km/s)	3.650	2.9	2.510	8.6	1.494	26.1
vS (km/s)	2.234	4.7	1.633	12.0	0.987	21.5
Ed (GPa)	31.6	6.8	15.8	19.9	5.6	52.7
vd	0.20	19.9	0.13	47.1	0.06	284
uD (GPa)	13.2	9.4	7.1	24.8	2.6	43.8
Kd (GPa)	17.6	11.3	7.2	14.8	2.6	69.9
K_IC_ (MPa√m)	0.90	18.4	0.45	14.2	0.16	16.1

In the table, the mean values (µ) are presented together with corresponding relative standard deviations (σ), where available, expressed as a percentage of the mean value.

To capture the variability of mechanical properties more comprehensively, three specimens were analysed for each rock sample. Among the properties evaluated, porosity demonstrates a pronounced discrepancy based on the granite’s state. In crystalline rocks, the relatively small porosity and its variability are primarily due to the presence of cracks, which exert a more substantial influence on mechanical properties. As anticipated and consistent with numerous other studies^56^, weathering leads to heightened porosity, resulting in a significant degradation of the mechanical properties. Considering Sample A as an intact rock, Sample B manifests around a 50% reduction in elastic moduli and Mode I fracture toughness, while the weathering evident in Sample C induces an 80% deterioration in its mechanical attributes. In the context of dynamic methods (seismic velocities and corresponding elastic moduli), a notable increase in standard deviations linked to rock degradation is observable (Table 1). Conversely, variations in KIC are essentially independent of the granite’s condition. This disparity could potentially arise from the dynamic test’s heightened sensitivity to heterogeneities when compared to static KIC loading. Figure S3 illustrates weathering-induced dissimilarities in mechanical behaviour as observed during KIC tests. With increasing weathering levels, a decline in peak load, elasticity (slope of force/displacement pre-peak), and rock brittleness (force decrease post-peak) are evident. On the other hand, from Sample A to Sample C, the rock demonstrates a more plastic response around the peak load.

### Post-processing of laboratory thermal images

**Figure 3:**
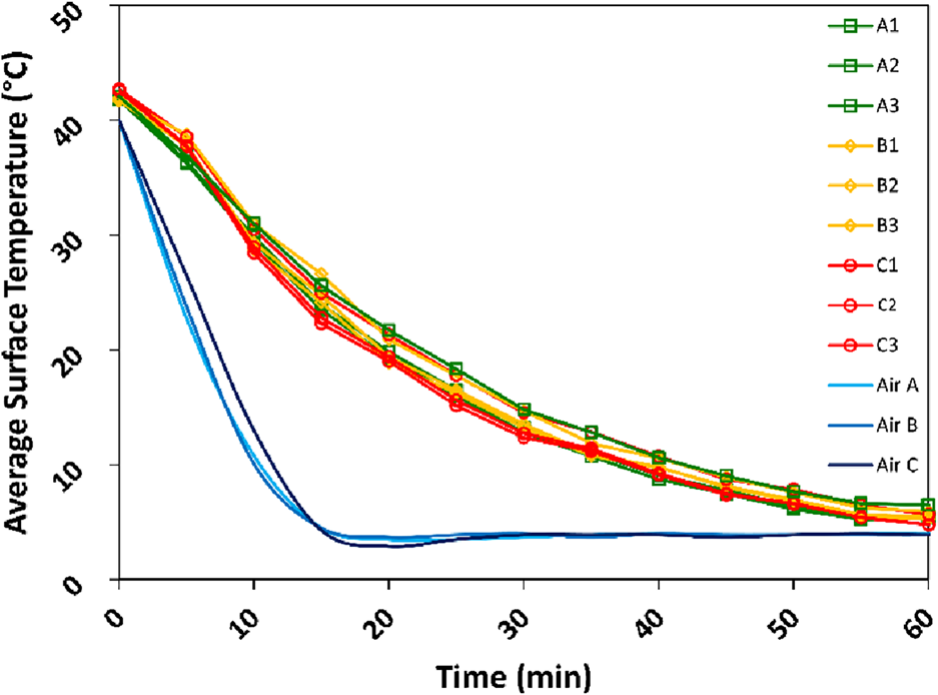
*IRT* time-lapse monitoring of cooling phases of cored samples collected at the *ROIs* and analysed in a climate chamber. Air A, B and C refer to the chamber’s air temperature throughout the three distinct experiments.

A laboratory-based *IRT* time-lapse was conducted on the cored samples collected at the *ROIs* to permit a comparison with the cooling patterns in the field. Given the notable differences in size and time duration between the field *ROIs* and the laboratory samples, a concentrated cooling experiment was performed (Fig. 3). The experiment encompassed a temperature range from 40 °C to 4 °C. The choice of 4 °C as the lower limit was intentional, stemming from the characteristic behaviour of liquid water to attain its highest density at this point. Beyond this threshold, water expands slightly until it reaches its freezing point, a phenomenon undesirable for the purposes of the experiment.

The climate chamber served as the controlled environment for this test, in which both humidity and temperature were monitored throughout the duration of the experiment. Prior laboratory tests established that prolonged experiments render CRI results not immediately obvious. Thus, having recognised the unfeasibility of achieving an exact comparison between the two cooling phases (in terms of time and amplitude), a method was implemented to seek insights that would be usable in practice. Thermal variations in laboratory tests are, in fact, induced by the air temperature. In contrast, in the field, sun radiation is the main energy source. Nevertheless, cooling is produced not only by the thermal conductivity between rock and air but also by radiation of the rock surface, and the difference between laboratory and field is related to that to some extent (see “Results and Discussion”).

### Comparison of cooling rate indices between field and laboratory

With the underlying premise that the same rock material and degree of weathering would yield a similar thermal response in both field and laboratory tests, an exploration of CRIs for varying time intervals (i.e., 10, 20, 30, 40, 50, 60 min) beginning from the peak temperature was undertaken. The cooling phases were analysed following Loche et al.^12^ and Mineo and Pappalardo^15^ for the field and the laboratory, respectively. Both of these are based on the evaluation of the release of the internal heat of rock blocks and samples and the evaluation of possible correlations between the CRI and internal properties of the materials. Subsequently, the various CRIs were plotted against porosity, a key parameter indicative of the rock’s weathering state^57^. Pearson’s correlation coefficients were then computed to determine the optimal fit and select the most suitable CRI interval for Young’s Modulus, Shear Modulus, Poisson’s ratio, and KIC. By utilising the CRI interval that yielded the best performance, along with the shortest period, correlations with these parameters were established.

The laboratory experiments were interpreted through linear relationships. To validate the robustness of these relationships, a Bayesian regression analysis was conducted utilising the *rstanarm* package (https://mc-stan.org/rstanarm/) within the R environment (Fig. S4). Subsequently, a linear regression model was established employing CRI values from the laboratory as predictors for porosity, as delineated in Eq. (2):

2 $$ \mu n \, = \beta_{1} X_{i} + \, \beta_{0} . $$

In the specified equation, *µn* represents a particular porosity value, while the model coefficient *β*_*0*_ serves as the intercept denoting the porosity at which the CRI was 0 (*X*_*i*_ = *0*). *β*_*1*_, referred to as the CRI coefficient, signifies the alteration in porosity corresponding to a CRI increase, while the *X* coefficient in the model is equivalent to a slope.

We ran the regression analysis using a Bayesian regression modelling approach, where we did not acquire single point estimates of coefficients but the entire distributions of simulations that represent possible values of the coefficients given by the model. In other words, the possible linear relationship between the data inputs and their variability predicts a series of coefficients which describe the relationship between the predictors and the outcome variable. In the specific case, the regression models were configured to generate 10,000 simulations from the posterior distribution for each model parameter. Subsequently, we extracted these 10,000 simulations from the model results and graphically displayed the principal statistical parameters in Fig. S4. The figure demonstrates an equation remarkably akin to that obtained from the laboratory test results, thereby affirming the robustness of such a relationship. Therefore, the data from field and laboratory tests can be interconnected, thereby enabling the use of the physical insights garnered from laboratory experiments to evaluate the porosity or other physical properties by means of CRIs. However, further tests are necessary to validate the present findings. Thus, to exemplify this strategy and to allow reproducibility, a flowchart has been included in Fig. S5.

## Results and discussion

**Figure 4:**
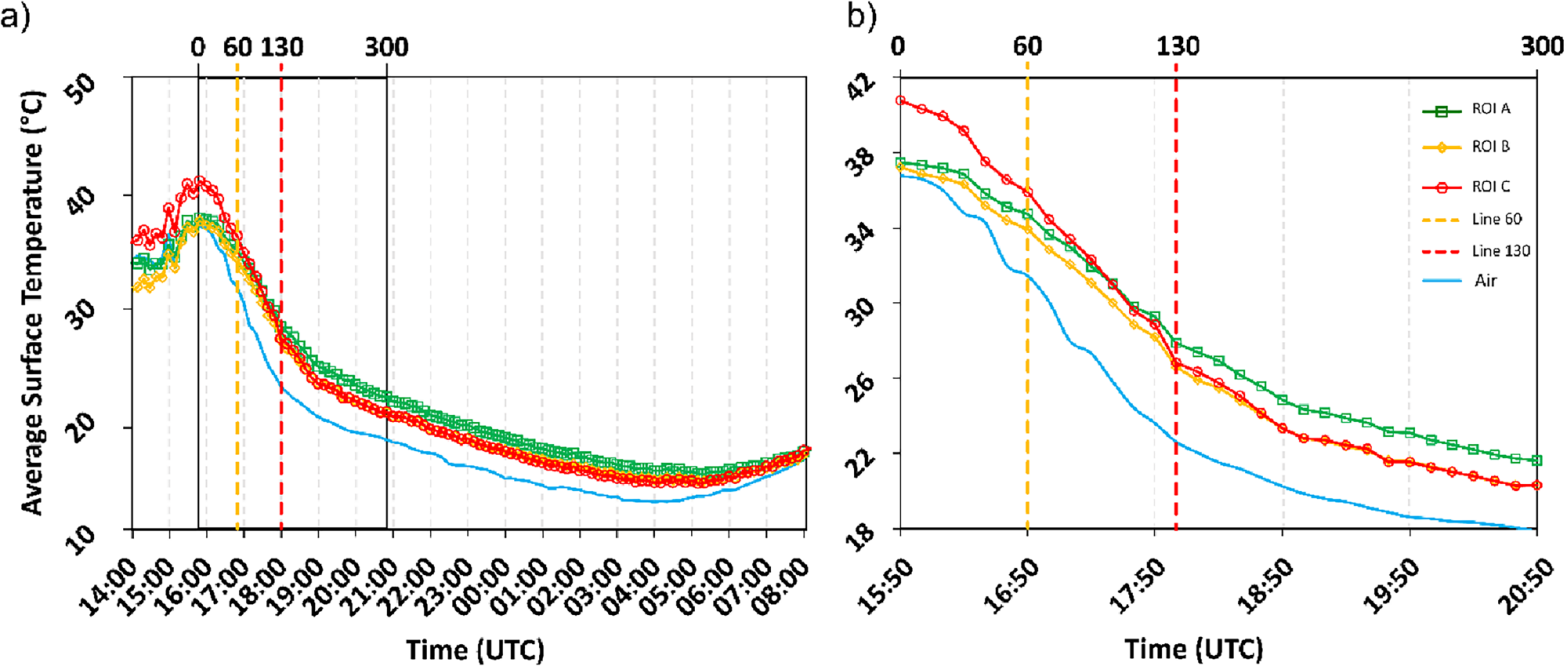
(**a**) Cooling phase of the three *ROIs* (A, B, C) after the peak temperature (15:50) till the lowest temperature; (**b**) detail of the first four hours (300 min) of the cooling phase and the time of the inflection point for all the *ROIs*, reached at 18:00.

**Figure 5:**
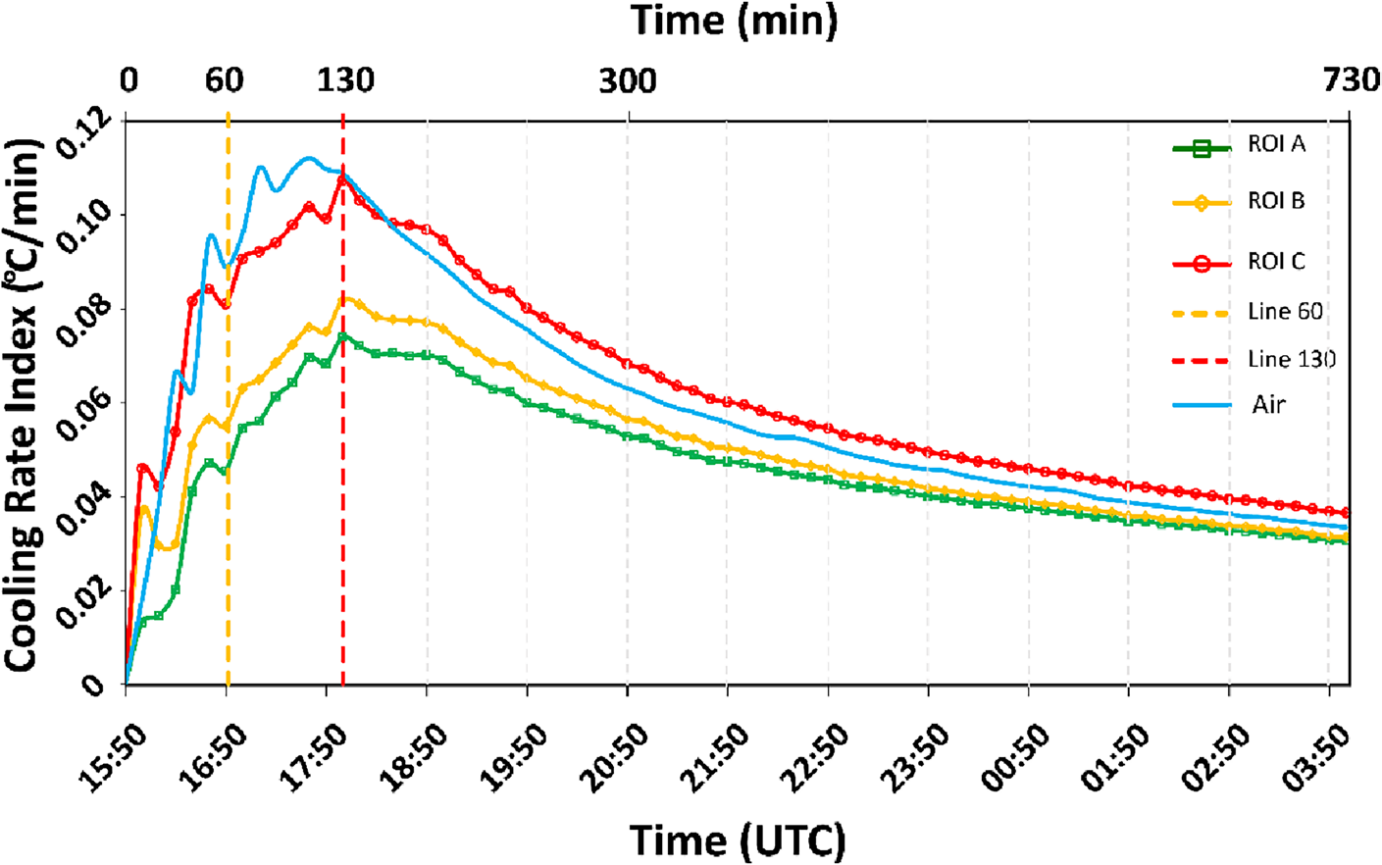
Changes in CRIs, computed on a 10-min basis from the beginning of the cooling (15:50) at the different *ROIs*. The red-coloured dashed line shows the *ITW* or *inflection point* after which the use of CRI progressively yielded useful and comparable information in the field. Especially, the red line signs the peak in CRI values after 130 min (18:00) from the peak in temperature, while the yellow line signs the end of 60 min as a straight comparison with the laboratory tests from the peak in temperature. The definition of *ITW* for a longer period after the inflection has a rigorous mathematical basis, established by the progressively linear decrease of CRI values (Fig. S6).

The field *IRT* time-lapse monitoring (Fig. 4) indicates a peak air temperature of 36.77 °C at 15:50 (all times are in UTC). Temperature within the selected Regions of Interest (*ROIs*, see “Methods”) peaked at 15:50 as well, with values of 37.47 °C, 37.23 °C, and 40.78 °C in *ROI* A, B, and C, respectively. The lowest air temperature (12.33 °C) was attained at 4:00, whereas the lowest temperature within the *ROIs* was recorded at 5:10–5:20, with values of 14.81 °C, 14.18 °C, and 13.98 °C for A, B, and C, respectively. The time interval between the maximum and minimum temperatures defines the limits for the evaluation of the CRIs. In Fig. 4, it can also be seen that the more weathered *ROI* C attained the highest temperature and also exhibited the most rapid cooling. Conversely, *ROI* A (intact rock) attained a lower peak temperature and underwent slower cooling. These behaviours may emerge from concurrent processes, possibly at least partly resulting from the different porosities of the *ROIs*. Higher porosity (with air-filled pores) means lower heat capacity and thermal conductivity but, at the same time, better convection if the pores are interconnected. Consistently, *ROI* C also demonstrated a propensity to lose stored internal heat more swiftly during cooling in comparison to the other two *ROIs*. To better examine these patterns, the CRIs between 15:50 and 4:00 were plotted at 10-min intervals as in Fig. 5, which shows the peak CRIs occurring at 18:00, ~ 130 min after the beginning of the cooling phase. Then, the CRIs decreased for all *ROIs*, paralleling the decline in the CRI of air temperature. Accordingly, the dissimilarities in cooling behaviour among the distinct *ROIs* gradually diminished. This evolving pattern carries significant implications, with the initial segment of the cooling phase emerging as the most informative sector for evaluating the thermal characteristics of various portions of the rock mass.

**Figure 6:**
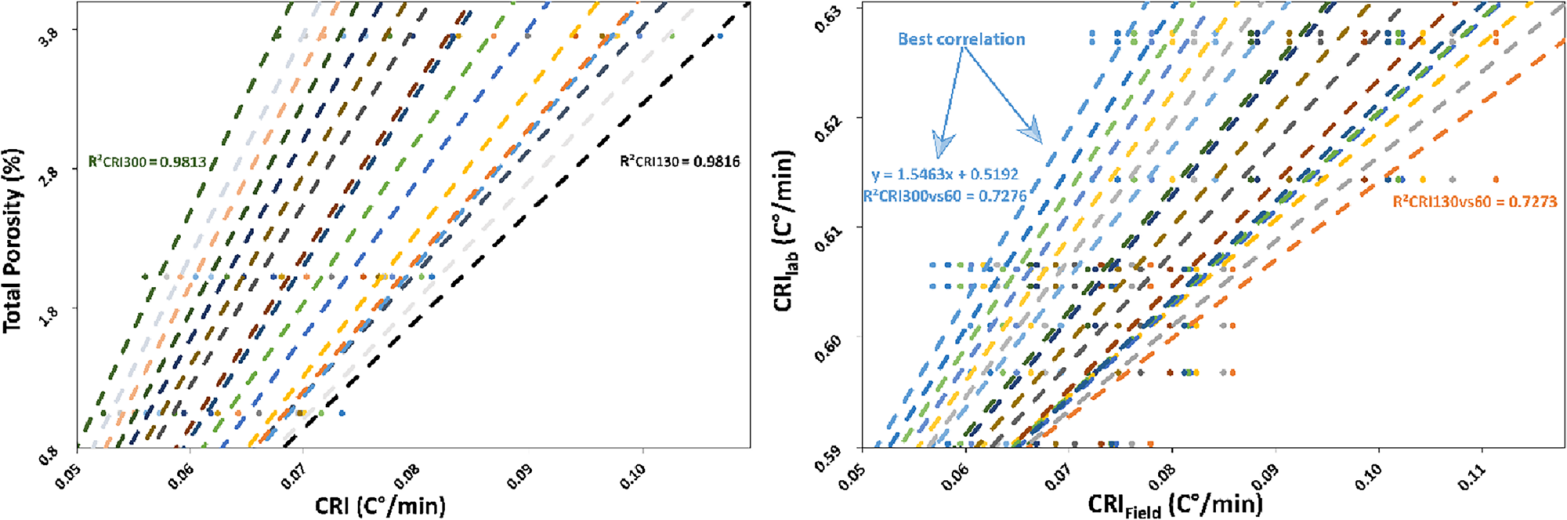
(**a**) Linear regressions of CRIs and porosity in the field. It corresponds to the values along time series CRI(t) from t = 130 min to t = 300 m after peak of temperature; (**b**) comparison of field and laboratory results in the 130–300 min interval for the field. The best correlation was found between CRI_lab60min_ and CRI_field300min_. CRI for laboratory samples is computed uniformly for t = 60 min, which revealed the best correlation.

The Informative Time Window (*ITW*) is herein defined as the segment of the cooling curves starting at the temperature peak and extending until the conclusion of cooling. As illustrated in Fig. 6a, the convergence of the CRI curves of the different *ROIs* towards the highest positive values (130 min) upon entering the *ITW* enhances the potential to distinguish and compare differences between field and laboratory results. It is important to note that the optimal *ITW* might differ based on factors such as lithology, slope orientation, and seasonal variations. In a coastal cliff study by Loche et al.^12^, the most suitable interval for achieving the highest correlations in rock strength was found to be 300 min. This *ITW* was identified during a summer campaign in Sardinia, Italy, on a southeast-facing granite slope. Furthermore, Pappalardo et al.^39^ observed significantly faster cooling during *IRT* summer campaigns compared to winter campaigns, suggesting a correlation between temperature gradient and the informative period which is rooted in the actual physical process of heat transfer. Similar findings were observed in laboratory tests on compacted soil samples by Loche et al.^58^. In these cases, laboratory tests conducted at higher temperature gradients highlighted clear and rapid cooling phases, thus accentuating the possibility of estimating the intrinsic properties of the geomaterials.

These considerations underscore the importance of selecting appropriate time windows for meaningful results and advocate for laboratory tests that capture the most informative phase of cooling, typically around the peak of CRIs. In this regard, an inflection point on the curves at 18:00 in Fig. 4b indicates a transition to a slower and more uniform cooling process. This phenomenon, influenced by the difference between ambient and rock temperatures in the absence of direct solar insolation, is known to induce a gradual cooling phase in which the curves become parallel^15,16^. It remains to be determined whether the identified *ITW* is universally applicable across various materials with the same temperature range, or if it is specific to the investigated area. For instance, after defining the *ITW*, different CRIs were selected to clarify possible correlations.

**Figure 7:**
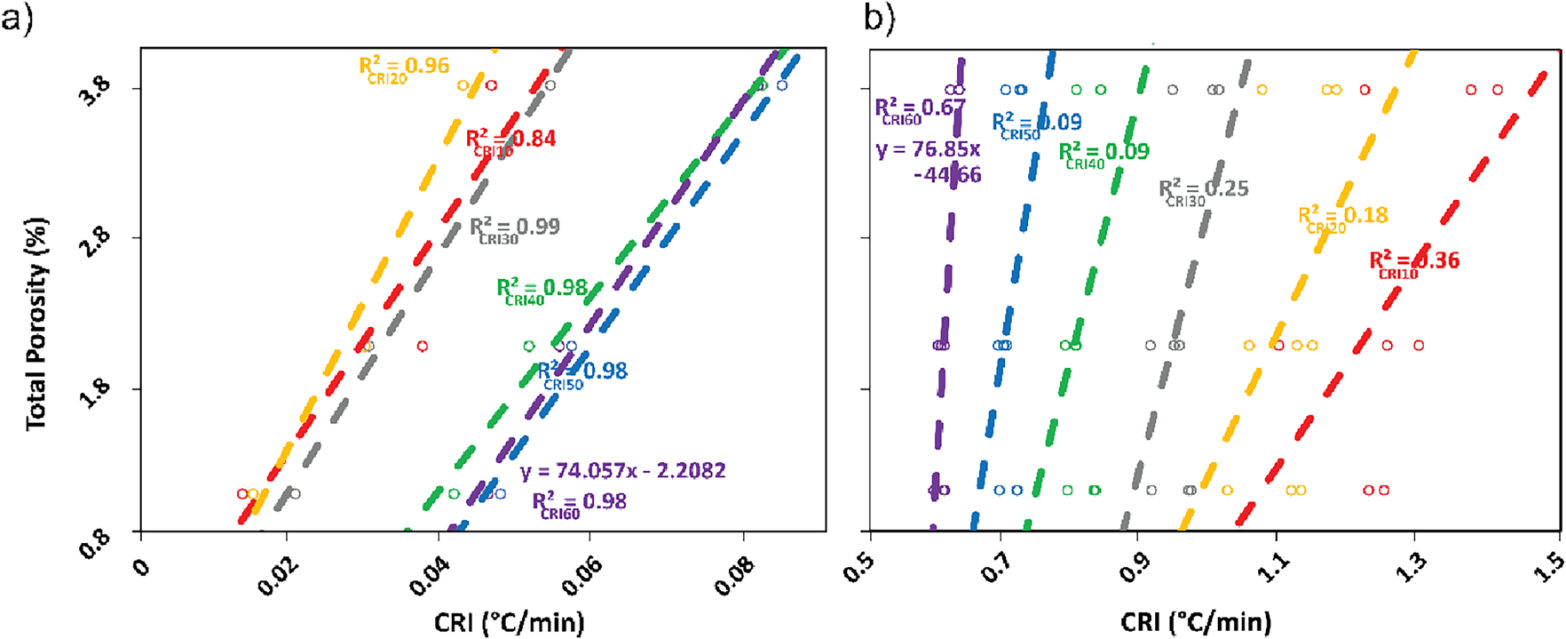
Cooling rate indices vs. porosity for (**a**) the field and (**b**) the laboratory.

As shown in Fig. 7a, CRIs for different time intervals plotted against porosity reveal that the slope of correlations changes over time. The highest correlation coefficient was achieved within the initial 30 min (R^2^ = 0.99) and 60 min (R^2^ = 0.98). Beyond 170 min, corresponding to the threshold of 130 min (*ITW*), correlations continue to yield reasonable outcomes (R^2^ > 0.8). Table S1 provides Pearson’s correlation coefficients and slopes for the various CRI intervals under examination. Notably, after 130 min, the slope of the CRIs diminishes, indicating a slower rate of change along the regression line. Given the well-established connection between temperature and porosity, particularly in laboratory tests, we contend that the most informative time window lies in the early stages of cooling. This assertion aligns with prior hypotheses put forth by Mineo and Pappalardo^15,16^. Similarly, laboratory tests conducted on Basalts, Calcarenites, Limestones, Quartzarenites, and Quartzsiltites^16^ and limestone rocks^59^ also pointed out the optimal utility of the early cooling phase. However, the shift from laboratory to field and the introduction of different temperature gradients yielded divergent outcomes, where time spans of 30–60 min proved to be sufficiently informative. Nonetheless, the intricate nature of the natural environment necessitates the selection of optimal conditions, including bright sunny days and elevated temperatures, to ensure robust *IRT* campaigns. In the field, as mentioned, the cooling process is complex, as the surrounding materials have also been exposed to solar radiation, and they are cooling as well and are radiant. Although each point of interest may be affected from different solar radiation, this is not the case in our study, where the vertical cliff should allow for a spatially homogeneous heat balance. Moreover, the altitude of the ROIs is negligible and should not affect the heat balance. Accordingly, the use of CRI_30min_ and CRI_60min_ for establishing empirical relationships was considered.

**Figure 8:**
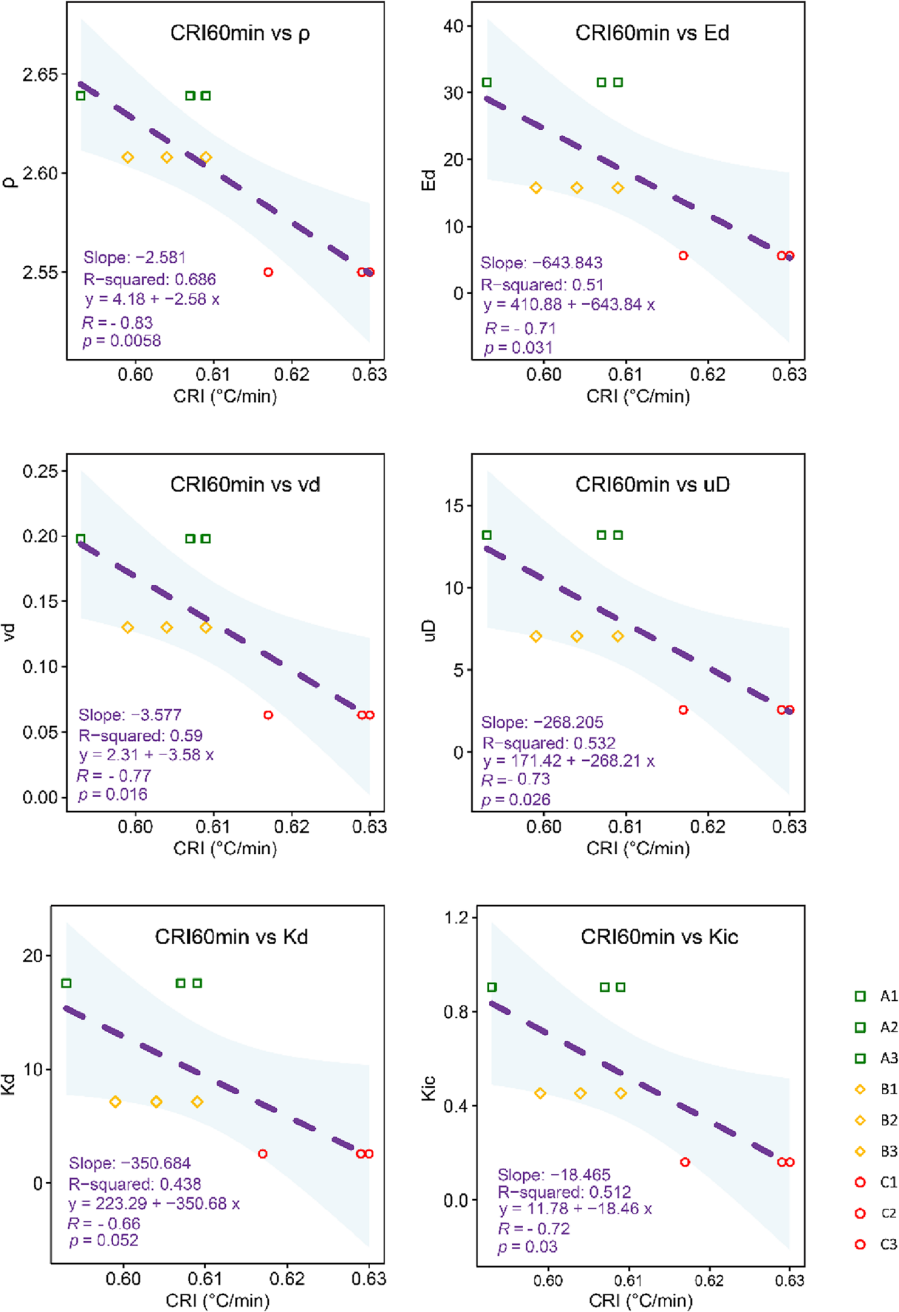
Regressions between CRI_60min_ and rock sample properties.

The empirical relationships were further extended to Young’s and shear moduli, Poisson’s ratio, and Mode I fracture toughness (Fig. 8). The most robust correlation was found between CRI_60min_ and the Poisson ratio. This is probably given by the relationship between variables. In fact, as porosity increases (more void space), Poisson's ratio tends to decrease. This is because in more porous rocks, the ability of the rock to deform laterally without significant axial strain is enhanced. These results highlight the relationships between CRIs and key elasticity parameters, which play a key role in constitutive modelling formulations^60^. Especially, these models are often founded on laboratory results, which may not fully account for the heterogeneity of field conditions.

Despite the correlations, the challenge at this stage lies in reconciling the experiments conducted in the field and laboratory to facilitate a comparative approach (Fig. 7). Supported by previous research, we decided to plot the whole linear regressions for the remaining period following the 60-min direct comparison period in the field experiment. Notably, the field test data began to align with the experiments shown in Fig. 7b. After 130 min, a reversal in the pattern of CRIs emerged, characterised by a progressive change in the slope of regression lines, similar to the same slopes evaluated from laboratory experiments (Fig. 6a).

The result of this attempt suggests the possibility of a direct comparison between the two experiments. As previously discussed, the *ITW* is defined as the period following the peak CRI value. Having identified the *ITW* during which the experiments exhibit congruent behaviour, we can proceed to search for two linear regressions, one derived from the laboratory (acting as the reference) and the other from the field experiment. Figure 6b shows the regressions, and it becomes plausible that the use of CRI_60min_ may serve better the purpose. The reliability of this correlation is supported by its derivation from controlled experiments conducted in a climate chamber, which ensures a high level of control over boundary conditions. It is also cautious to consider the integration of a longer CRI interval to encompass a more extensive and linear cooling phase, as previously mentioned. Therefore, 60 min is shown to be an acceptable time frame for cooling a sample and retrieving its thermo-mechanical behaviour. This assertion is also reinforced by the equation’s notable resemblance to findings presented by Pappalardo et al.^39^.

In Fig. 6b, we illustrated the relationship that closely aligns CRI_field_ and CRI_lab_, as described by the following equation (Eq. (3)):

3 $$ ce \, = 1.5463CRI_{field300min} + \, 0.5192. $$

Using *ce* and substituting the value of CRI_field300min_, we can use *ce* in the reference equation for CRI_lab60min_ (Eq. (4)).

4 $$ n\left( \% \right) \, = 76.856CRI_{60min} - \, 44.664. $$

**Figure 9:**
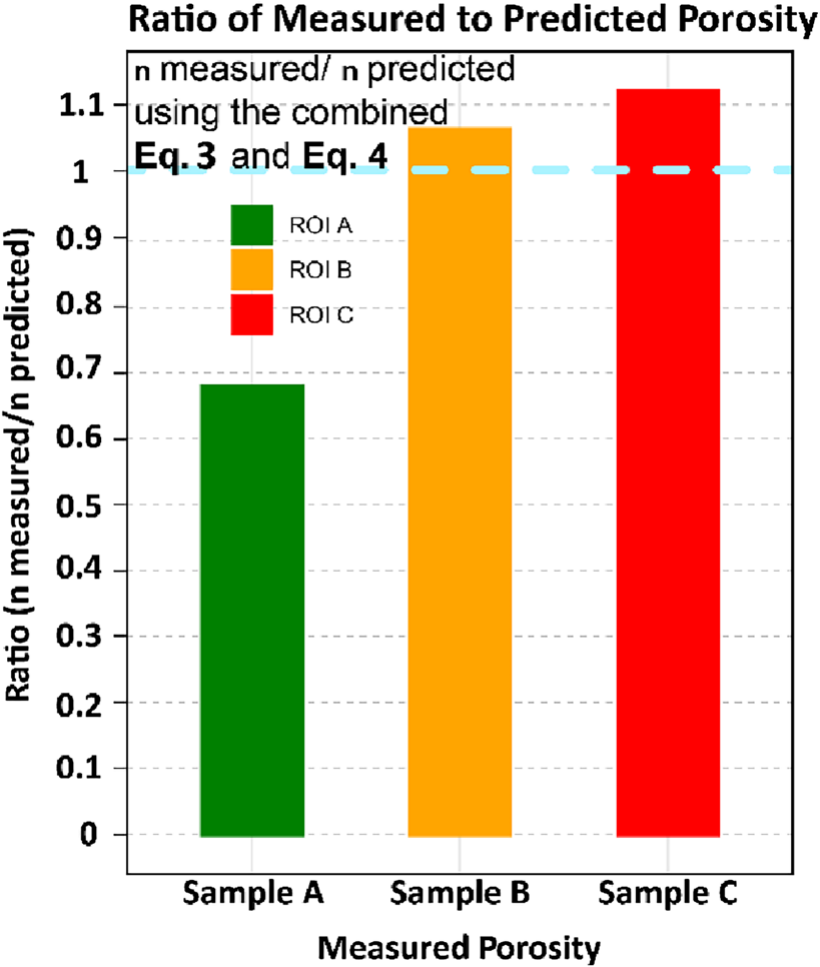
Performance of measure porosity in the field and predicted porosity from the method proposed (n_measured_ / n_predicted_) using a combination of Eqs. (3) and (4). The performance is plotted as a ratio, and values close to the unit represent predictions fitting the input values.

To estimate the goodness of the procedure, we attempt to derive the porosity value from the field CRIs. In a general approach, by combining Eqs. (3) and (4), we can assess the predictive capacity of the empirical equation by inputting the CRI values obtained from the field *ROIs* (Fig. 9). The figure illustrates a satisfactory prediction of the porosity for *ROI* A, B, and C, utilising the laboratory-based and supposed calibrated empirical equation. In the case of sample A, the measured porosity is very low, which might be associated with an inherent error in its measurement. This could consequently lead to a lower ratio compared to samples B and C. However, even with this issue, the ratio is close to the unit, thus representing a satisfactory prediction. Comparable outcomes can be investigated using a statistical approach within a Bayesian framework (Figs. S7, S8). This highlights the efficacy of *ce* in correcting the predictive capacity of Eq. (4). Additionally, Eq. (3) functions as a filter for scale and time factors, enabling a direct comparison of the two experiments. This comparison is only possible by evaluating the entire cooling phase of the field test and identifying the moment, post-peak in CRI, at which the two experiments converge in terms of slope and intercept.

While establishing a robust empirical correlation database for different rock material properties remains necessary, the work does identify valuable relationships for elastic and porous material properties that could be integrated into modelling frameworks.

## Conclusion

This study proposes some highlights about the potential of Infrared Thermography (*IRT*) in identifying weathered sectors within a rock mass. The challenge lies in achieving the best possible fit within a limited time span while attempting to compare field and laboratory results. This is particularly vital for designing effective field surveys given the complexity of external conditions.

The definition of the Informative Time Window (*ITW*) was crucial in narrowing down the interval of informativity. It was demonstrated that within the cooling phase, a shorter interval can yield better results, despite the challenge of establishing an identical relationship between hourly Cooling Rate Indices (CRIs) in both field and laboratory conditions. The study’s findings align with other authors' suggestions, encouraging summer surveys, significant temperature gradients, and variable *ITW*.

The introduction of a “comparison equation” (*ce*) played a pivotal role in normalising the results, accommodating differing temperatures and confined spaces, thus enhancing the predictive capacity of Eq. (4) for estimating porosity within the studied *ROIs*. While it may be specifically valid for this study, the approach shows feasibility and the potential to be applied to other rock types in similar conditions. The study’s ultimate objective was to establish an approach that could contribute to the remote characterisation and estimation of rock slope stability for modelling purposes using *IRT* campaigns. In this perspective, being *ROIs* boast high-resolution CRI values (or cooling phases) spatially distributed across the rock outcrop, such an approach demonstrated to potentially reduce analysis costs, expedite survey processes, and provide results closely aligned with field conditions.

However, it is important to emphasize that these findings are specific to the rock type and test site at Požáry, Czechia. The authors encourage the application of this method in diverse locations and lithologies to uncover additional empirical correspondences between temperature (cooling phase) and mechanical properties.

We used a single circle to plot the point prediction from the regression coefficients, and histograms to display the posterior distributions produced by the linear prediction with uncertainty (*posterior linpred*) and posterior predictive distribution (*posterior predict*). The plots show the level of uncertainty for each prediction. The point prediction is represented by a single value (yellow, orange and red). The linear predictions with uncertainty, which consider the posterior distributions of intercept and temperature coefficients, have pronounced peaks, whereas the model estimates vary within a limited range. On the other hand, the posterior predictive distribution varies much more. We used a Bayesian analysis to show that there is not a single prediction from a model for a given observation, but rather distributions of predictions. Overall, the above analyses quantify the model uncertainty and may help an expert judgment on the use of porosity prediction from CRI values.

### Supplementary Information

Supplementary Information.

Supplementary Video 1.

## Data Availability

Elaborated data are presented in the manuscript. Raw experimental data can be provided by M. Loche (loche@irsm.cas.cz) upon reasonable request.
