# Supplementary material for: Infrared thermography reveals weathering hotspots at the Požáry field laboratory
Source: Sci Rep. 2024 Jun 25;14:14682. doi: 10.1038/s41598-024-65527-x (PMC11199624; doi:10.1038/s41598-024-65527-x)
Supplement: Supplementary file 1 — Supplementary Information. [file 41598_2024_65527_MOESM1_ESM.docx]

Supplementary information for:

**Infrared thermography reveals weathering hotspots at the Požáry field laboratory**

Marco Loche^1,2^, Ondřej Racek^1,3^, Matěj Petružálek^1,4^, Gianvito Scaringi^2, *^, Jan Blahůt^1, *^

^1^ Institute of Rock Structure & Mechanics, Czech Academy of Sciences, V Holešovičkách 41, 182 09 Prague, Czechia

^2^ Institute of Hydrogeology, Engineering Geology and Applied Geophysics, Faculty of Science, Charles University, Albertov 6, 128 43 Prague, Czechia

^3^ Department of Physical Geography and Geoecology, Faculty of Science, Charles University, 128 43 Prague, Czechia

^4^ Institute of Geology of the Czech Academy of Sciences, Prague, Czechia

* Corresponding authors: gianvito.scaringi.natur.cuni.cz, [blahut@irsm.cas.cz](mailto:blahut@irsm.cas.cz)


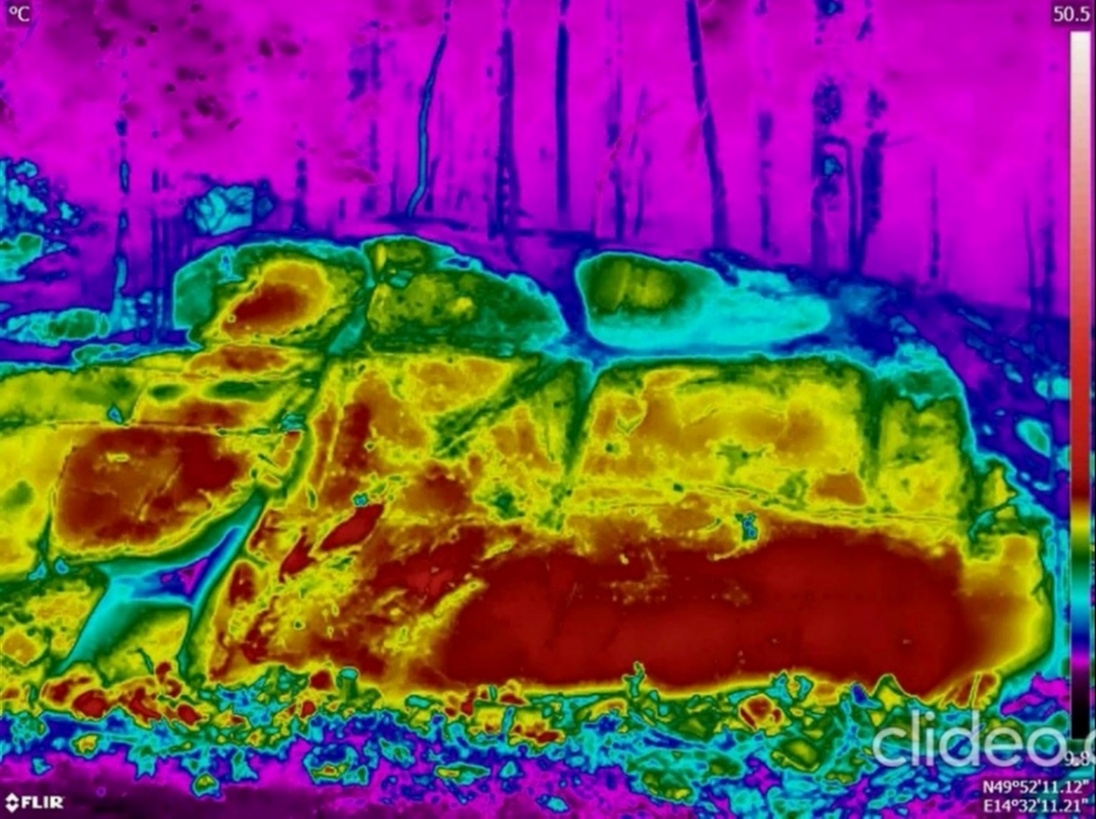


**Figure S1**. Example of snapshot from the time-lapse video available as supplementary material.


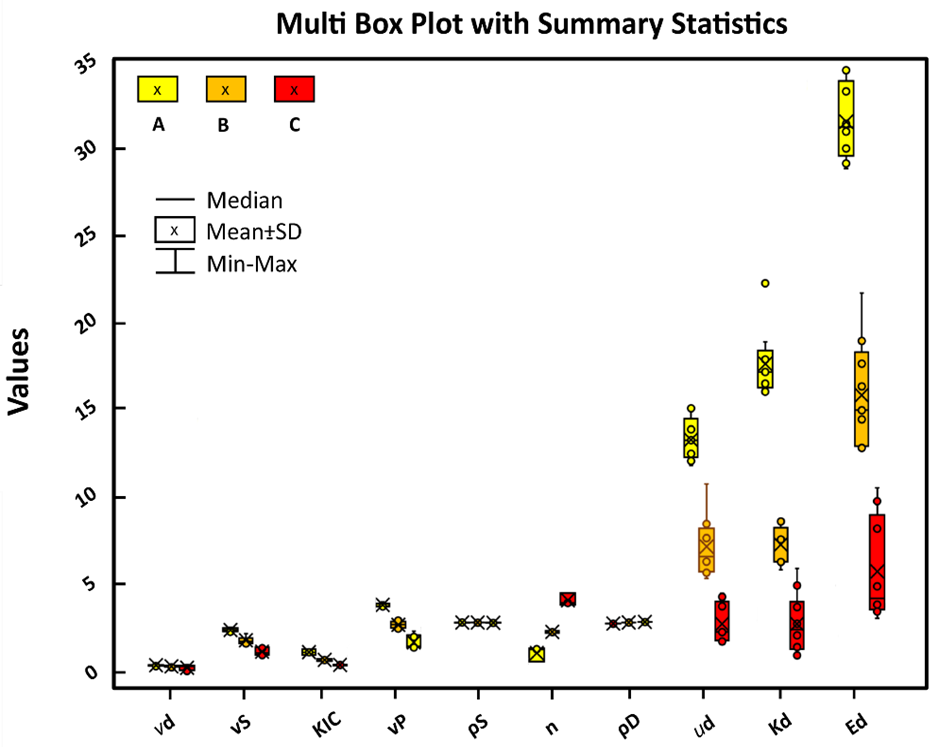


**Figure S2.** Results of laboratory tests: grain density (ρS); dry density (ρD); total porosity (n); P-wave velocity (vP); shear wave velocity (vS); Young’s modulus (Ed); Poisson’s ratio (ud); shear modulus (vd); bulk modulus (Kd); Mode I fracture toughness (KIC). The boxplots visualise the mean values and standard deviations.


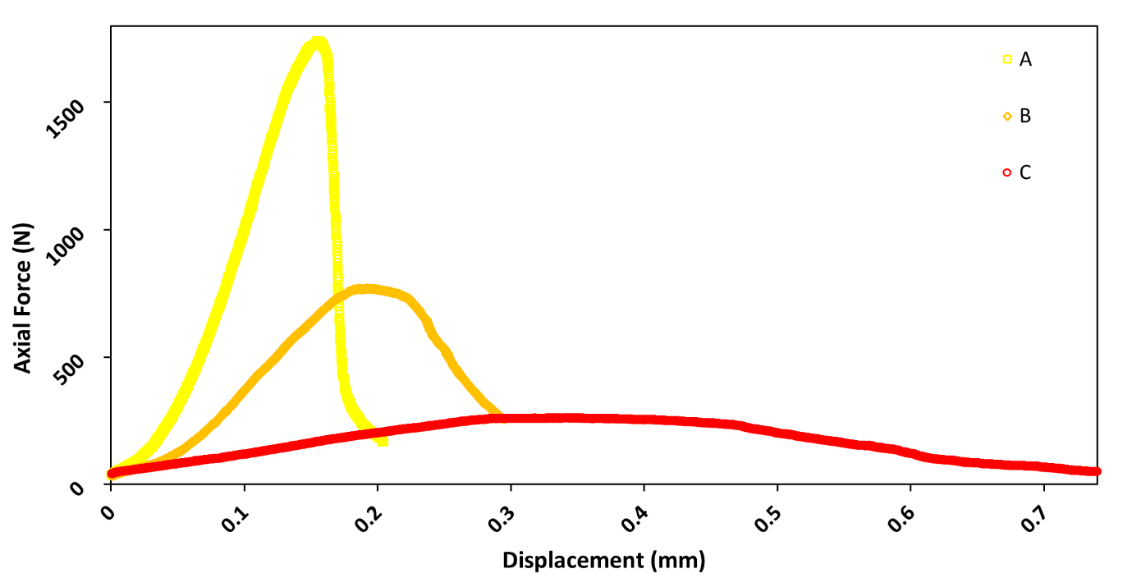


**Figure S3.** Characteristic differences in mechanical response during KIC tests related to the state of the samples A, B, and C. The measured axial force is plotted against the vertical displacement, which was used to control the loading during fracture toughness tests.


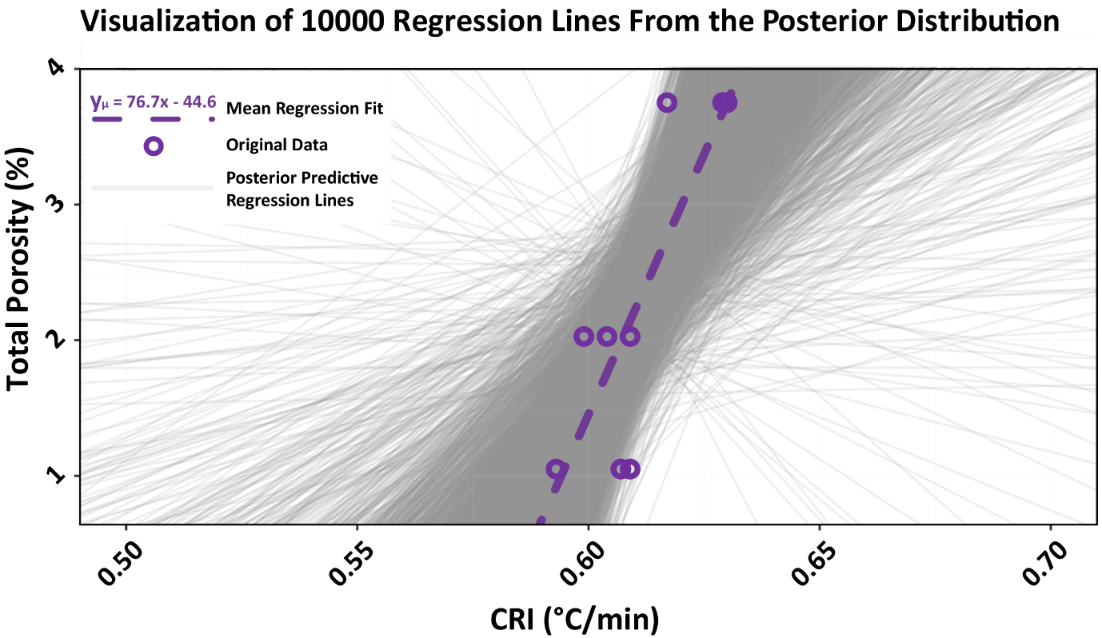


Figure S4. The graph shows 10,000 regressions, one for each simulation from the posterior distribution. In light grey, a sample of regression lines from the posterior distribution. In violet, we plotted the mean slope of all the posterior simulations (colour of the CRI60min vs. Porosity correlation). The plot displays the uncertainty about the size of this relationship and shows that some of the slopes from the distribution are negative. Structurally, we can observe a mean coefficient estimate and the Bayesian framework via the posterior distributions provided additional information about the uncertainty of our estimation. For the example of CRI_lab60min_, the model in the figure has intercept *β_0_*=−44.6 and slope *β_1_*=76.7. The slope is meaningful, indicating that for every increase in CRI of 0.025, we would expect an increment of 1.5 (%) in porosity.


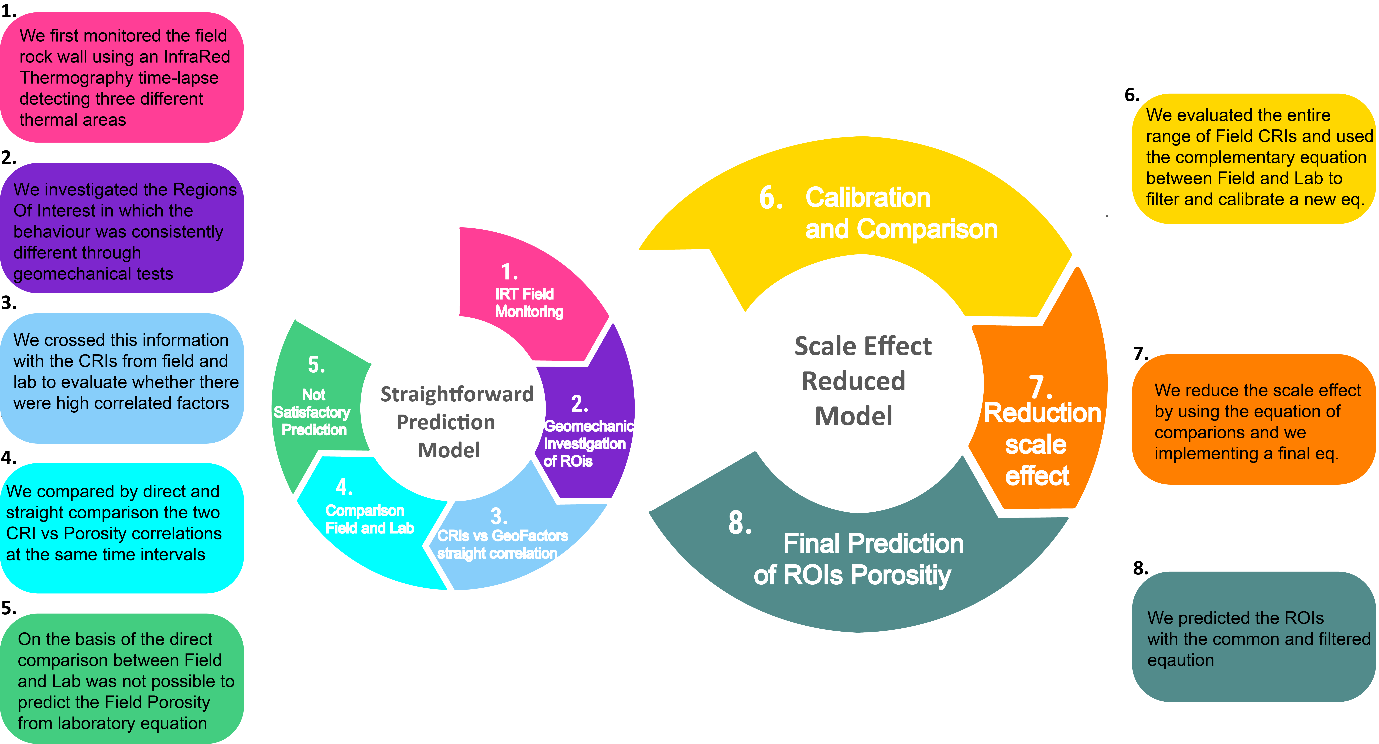


**Figure S5**. Flow chart summarising the steps of this research.


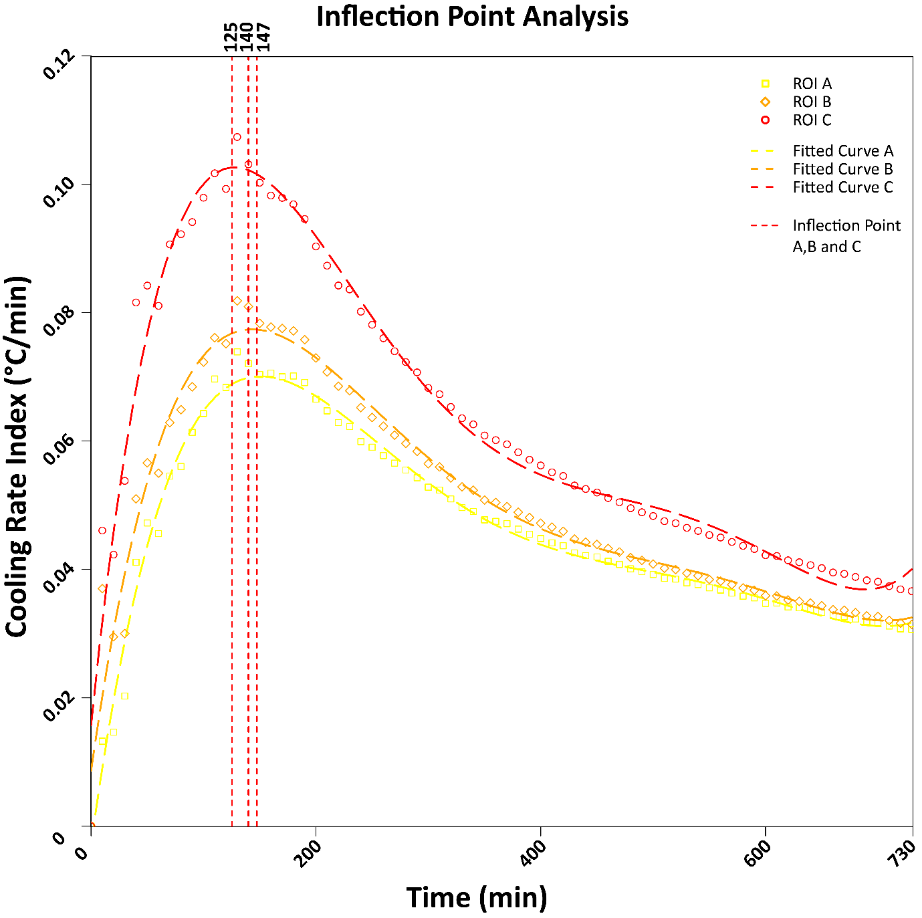


**Figure S6.** Identification of inflection points in the relationship between CRIs and time for field data using a polynomial model of degree 5. The figure plots the original data points of *ROIs* and the fitted curves and adds vertical lines (dashed red lines) at each inflection point.


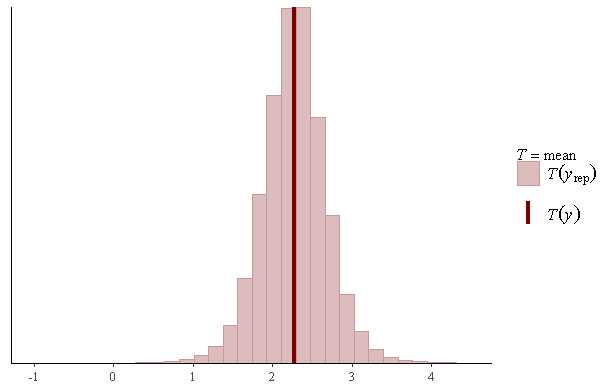


**Figure S7.** The graph shows that the mean value of the CRI variable, in the original dataset, falls in the centre of the posterior predictive distribution.


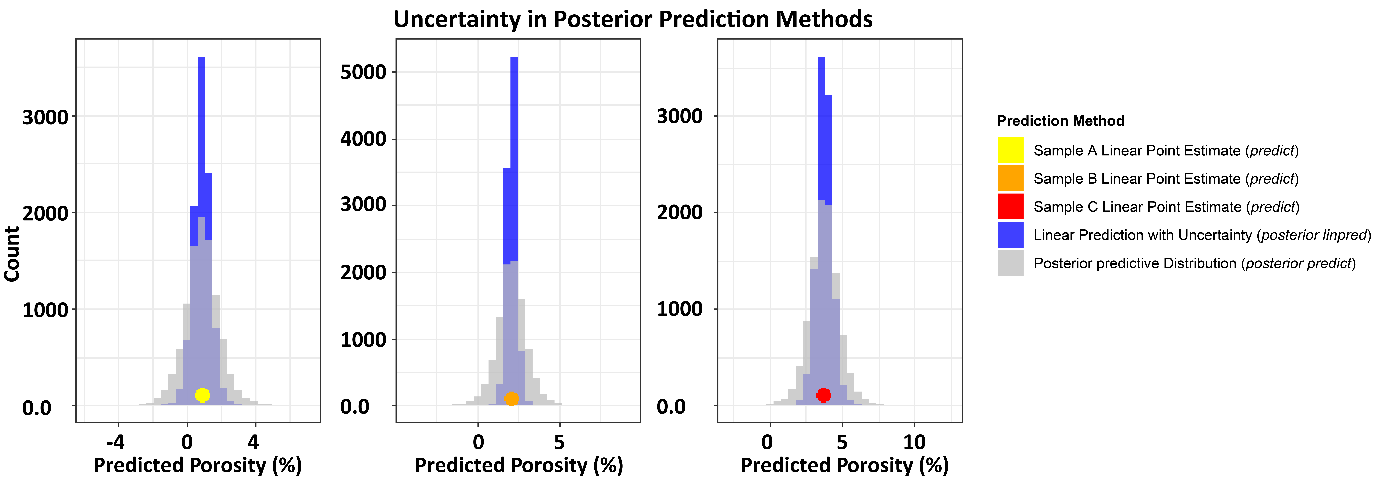


**Figure S8.** The graphs display the results of the predictions we made for sample *A* (result=0.891022; original=1.049868), Sample *B* (result=2.115057; original=2.027292) and Sample *C* (result=3.771731; original=3.752481), using respectively CRI_lab60min_a_, CRI_lab60min_b_ and CRI_lab60min_c_.

**Table S1.** Pearson’s correlation coefficients and slopes for different CRIs and rock sample porosity.

|  | CRI_30min_ | CRI_60min_ | CRI_90min_ | CRI_120min_ | CRI_240min_ | CRI_TOTAL_ |
| --- | --- | --- | --- | --- | --- | --- |
| Pearson | 0.997 | 0.994 | 0.988 | 0.988 | 0.994 | 0.970 |
| Slope | 0.013 | 0.013 | 0.012 | 0.012 | 0.008 | 0.002 |
